# Use of Tissue-Specific MicroRNA to Control Pathology of Wild-Type Adenovirus without Attenuation of Its Ability to Kill Cancer Cells

**DOI:** 10.1371/journal.ppat.1000440

**Published:** 2009-05-22

**Authors:** Ryan Cawood, Hannah H. Chen, Fionnadh Carroll, Miriam Bazan-Peregrino, Nico van Rooijen, Leonard W. Seymour

**Affiliations:** 1 Department of Clinical Pharmacology, University of Oxford, Oxford, United Kingdom; 2 Department of Molecular Cell Biology, Vrije Universiteit, Amsterdam, The Netherlands; Oregon Health & Science University, United States of America

## Abstract

Replicating viruses have broad applications in biomedicine, notably in cancer virotherapy and in the design of attenuated vaccines; however, uncontrolled virus replication in vulnerable tissues can give pathology and often restricts the use of potent strains. Increased knowledge of tissue-selective microRNA expression now affords the possibility of engineering replicating viruses that are attenuated at the RNA level in sites of potential pathology, but retain wild-type replication activity at sites not expressing the relevant microRNA. To assess the usefulness of this approach for the DNA virus adenovirus, we have engineered a hepatocyte-safe wild-type adenovirus 5 (Ad5), which normally mediates significant toxicity and is potentially lethal in mice. To do this, we have included binding sites for hepatocyte-selective microRNA mir-122 within the 3′ UTR of the E1A transcription cassette. Imaging versions of these viruses, produced by fusing E1A with luciferase, showed that inclusion of mir-122 binding sites caused up to 80-fold decreased hepatic expression of E1A following intravenous delivery to mice. Animals administered a ten-times lethal dose of wild-type Ad5 (5×10^10^ viral particles/mouse) showed substantial hepatic genome replication and extensive liver pathology, while inclusion of 4 microRNA binding sites decreased replication 50-fold and virtually abrogated liver toxicity. This modified wild-type virus retained full activity within cancer cells and provided a potent, liver-safe oncolytic virus. In addition to providing many potent new viruses for cancer virotherapy, microRNA control of virus replication should provide a new strategy for designing safe attenuated vaccines applied across a broad range of viral diseases.

## Author Summary

Attenuated viruses have found important applications in medicine, including their use as vaccines (notably for measles, mumps, polio, influenza, and chicken pox) and their experimental development as selective cancer-killing agents, so-called “virotherapy.” Wild-type versions are often most effective in both of these settings; however, attenuated viruses have usually been developed to decrease the risk of significant viral pathology. Recent advances in understanding regulation of gene expression by microRNA now afford the possibility to design viruses that are “selectively attenuated” in sites of potential pathology, by engineering them for inhibition by microRNA molecules that are expressed there. Here we have engineered wild-type adenovirus for recognition by a microRNA expressed in hepatocytes, producing a virus that retains wild-type infection and replication at sites of therapeutic activity (such as cancer cells) but is severely attenuated in hepatocytes, both *in vitro* and *in vivo*. This virus caused no significant liver toxicity to mice even when applied at ten times the lethal dose of wild-type virus. The ability to produce replication-competent viruses with key toxicities removed should provide a new platform for development of improved cancer treatments and better vaccines for a broad range of viral diseases.

## Introduction

Viruses have a highly successful history as prophylactic vaccines and are also being developed for their intrinsic anticancer activities [1]. In both settings the ability to undergo restricted replication is highly desirable. Attenuated (but not killed) viral strains often represent the most effective viral vaccines, affording the possibility of persistent low level infection without significant pathology [2],[3]. Unfortunately many viruses are not suitable for production of attenuated forms, and reversion to wild-type represents a significant risk. Equally the field of cancer ‘virotherapy’ relies on selective replication of lytic viruses within cancer cells, leading to cell death and spread of infection to adjacent cancer cells. Several ‘conditionally-replicating’ viruses have been engineered for activation by tumour-associated changes, showing greater potency in cancer cells than in normal cells. Unfortunately these agents are generally attenuated compared to the equivalent wild-type virus even in cancer tissues, and have so far shown little therapeutic activity in clinical trials [4],[5]. For both vaccination and cancer virotherapy it would be attractive to produce viruses that show wild-type replication activity at therapeutic sites (eg. within tumours or at sites of antigen presentation) but are specifically attenuated at sites of potential pathology.

The network of naturally-occurring non-coding microRNA molecules [6] negatively regulates cellular gene expression post-transcriptionally through a number of mechanisms that all involve binding of microRNA to complementary regions within a messenger RNA (mRNA) [7],[8] leading to decreased protein production [9]. Tissue-selective microRNA expression is now well characterised [10], and it provides an opportunity to regulate transgene expression from therapeutic nucleic acids and viruses. This principle was originally developed by Brown et al. [11], who showed that inclusion of microRNA mir-142-3p binding sites within 3′UTR of retrovirally-encoded transgenes prevented expression in antigen presenting cells, preventing stimulation of an immune response and allowing long term transgene expression in other cells without rejection. The diversity of tissue-specific microRNAs now identified should enable this approach of selective attenuation of viral expression to be developed in several different contexts [12].

It is also possible to use the microRNA system to regulate replication of vaccines or conditionally-replicating ‘oncolytic’ viruses. The small size of the required insertion (microRNA binding sites are generally only 21–24 bp) provides considerable flexibility in the design of capacity-restricted therapeutic viruses. Equally the negative regulatory principle of using microRNA regulation to inhibit viral replication in sites of toxicity, could allow significant therapeutic potency by avoiding attenuation in target sites. This approach has previously been used to generate microRNA-controlled conditionally-replicating RNA viruses, including Polio virus for vaccination and coxsackie virus for cancer virotherapy. These viruses were engineered to contain binding sites for neural and muscle specific microRNAs respectively. The neural-restricted polio virus showed good vaccine potential, while the muscle-restricted coxsackie virus showed decreased myositis and improved anticancer efficacy [13],[14].

In this study we have explored the use of this approach in engineering a microRNA-controlled wild-type adenovirus, a DNA virus, by expressing binding sites for microRNA mir-122 within the 3′ UTR of E1A. Mir-122 is highly and selectively expressed in hepatocytes [10],[15], and this modification might prevent expression of E1A within hepatocytes, thereby reducing adenovirus replication and hepatotoxicity whilst maintaining its therapeutic replication within tumour cells.

## Results

### Evaluation of potency of mir122 regulation *in vitro*

**Figure 1:**
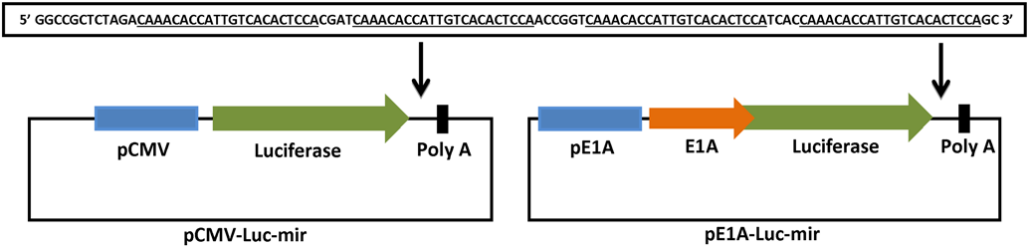
Plasmid construction. pCIK-Lux (referred to as pCMV-Luc) was cleaved with Not1 and concatamers of mir-122 binding sites (4 or 8 sense, or 4 antisense; the sequence of the 4 sense insert is shown at the top of the figure) inserted into the luciferase 3′UTR. Both pCMV-Luc and the version containing 4 microRNA sites (pCMV-Luc-mir) were modified with the C terminal half of E1A expression cassette, isolated from pAd5WT (Ad5 wild-type) by PCR. Both resulting constructs were then cloned into pAd5Kpn1, which contains the E1A promoter and coding sequence, to produce E1A promoter regulated E1A-luciferase fusion constructs termed pE1A-Luc and pE1A-Luc-mir.

**Figure 2:**
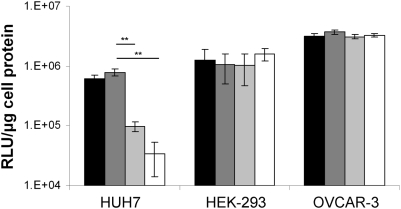
Effects of microRNA binding sites on expression of CMV promoter driven luciferase plasmids *in vitro*. Cells were seeded in triplicate in 12 well plates. After 24 h 0.5 µg of plasmid DNA (containing 0 (black), 4 (light grey) or 8 (white) sense mir-122 binding sites, or 4 antisense binding sites (dark grey)) was mixed with 2.5 ul DOTAP (Roche) reagent. 24 h following transfection cells were lysed and relative luminescence was measured using 25 µl cell lysate. N = 3, Error bars +/−standard deviation and data is shown as RLU/µg cell protein, determined by BCA assay. (** P<0.005).

To assess the repression capabilities of mir-122, CMV promoter-driven luciferase plasmids containing 0, 4 and 8 sense or 4 anti-sense microRNA binding sites (representative structures shown in Figure 1) were transfected into HEK-293, OVCAR-3 and HUH7 cell lines using DOTAP (Roche) and luciferase activity was measured by luminometry after 24 h. The presence of the microRNA binding sites had no effect on luciferase levels detected in the mir-122 negative cell lines HEK-293 and OVCAR-3 (Figure 2). In contrast, in mir-122-positive HUH7 cells, luminescence was decreased from 7.9×10^5^ RLU/µg (anti-sense control plasmid) to 9.9×10^4^ RLU/µg (4 microRNA binding sites, *P* = 0.001)) and 3.4×10^4^ RLU/µg (8 microRNA binding sites, *P* = 0.001). The inclusion of 4 anti-sense microRNA binding sites did not effect luciferase activity compared to the unmodified control plasmid in any cell type. Whilst the inclusion of 8 microRNA binding sites did show improved repression in comparison to 4 binding sites, we decided to use 4 binding sites for future use in view of the repetitive nature of the insertion and to minimise the likelihood of viral recombination.

### Evaluation of potency of mir-122 regulation *in vivo*

**Figure 3:**
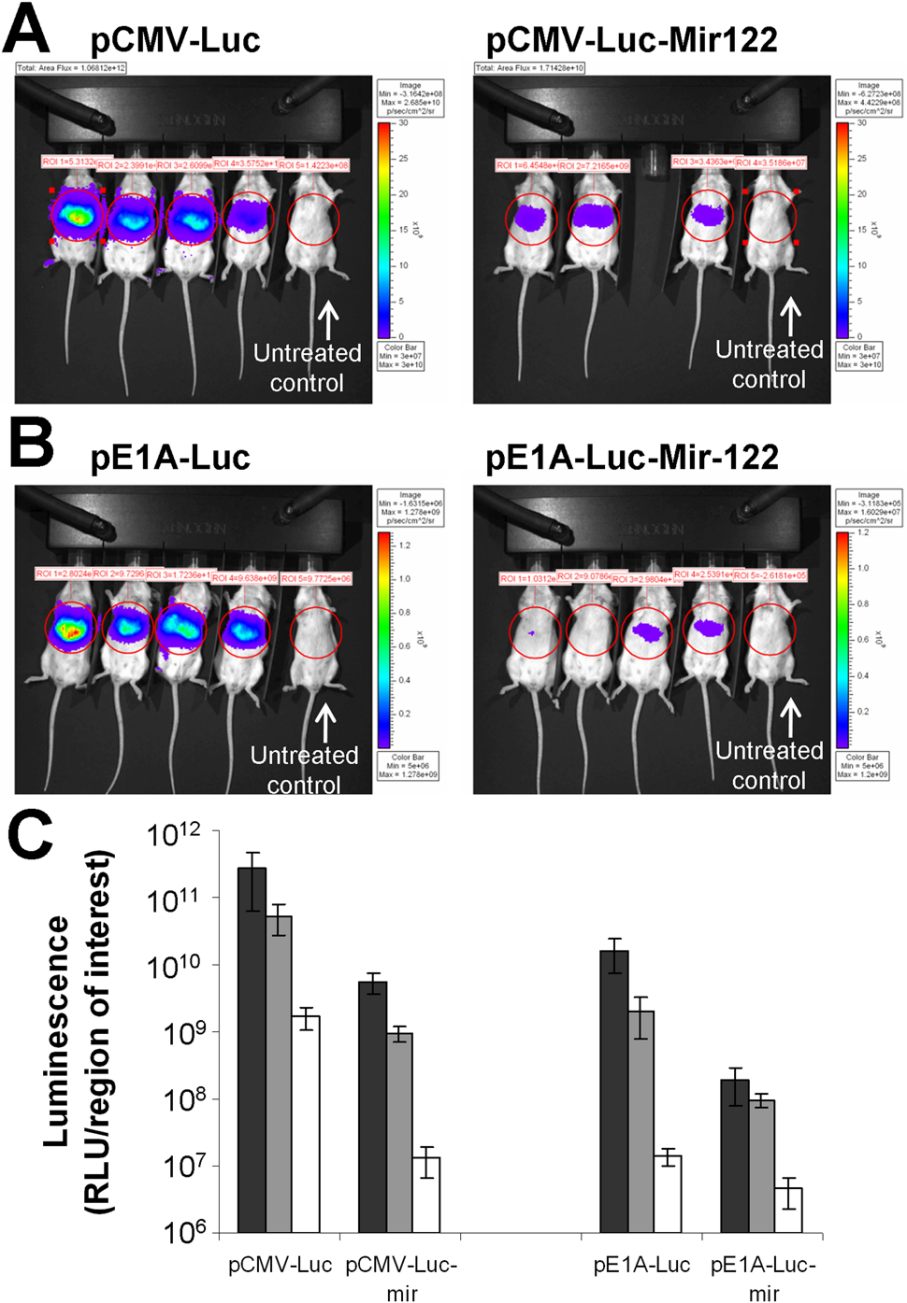
Effects of including binding sites for microRNA122a on expression of plasmids in vivo following hydrodynamic delivery to mice. (A) Imaging luminescence (8 h from mice administered pCMV-Luc not containing (left panel) and containing (right panel) four binding sites for mir-122 (plasmids pCMV-Luc and pCMV-Luc-mir in Figure 1).The animal on the right side of all images is a control treated with PBS, but mock injected with luciferin, used to provide a background reading. (B) Imaging luminescence (8 h from mice administered pE1A-Luc fusion constructs not containing (left panel) and containing (right panel) four binding sites for microRNA122a (plasmids pE1A-Luc and pE1A-luc-mir in Figure 1). The animal on the right is an untreated control. The two images in A are directly comparable with each other, as also the two images in B; however scaling is different between (A) and (B) in order to accommodate substantially different signal intensities from these plasmids. (C) Time course of luciferase expression from CMV promoter-driven and E1A promoter driven constructs shown in A and B. Black = 8 h, Grey = 24 h, White = 48 h. n = 4 throughout (except pCMV-Luc-mir122 where n = 3), error bars show standard deviation.

Luciferase expression from the microRNA-controlled plasmids shown in Figure 1 was assessed in murine livers *in vivo*, using an Ivis100 imaging system. Plasmid vectors were delivered at equimolar amounts by hydrodynamic delivery and imaging was performed at 8, 24 and 48 h post injection. Control CMV promoter-driven plasmids gave high levels of transgene expression after 8 h (2.7×10^11^ RLU) while inclusion of 4 microRNA binding sites in the same plasmid decreased expression to 5.7×10^9^ RLU, a 47-fold decrease in expression (Figure 3A). Total levels of luciferase expression fell substantially over the next 40 h, although the differential expression increased up to 129-fold (*P* = 0.0064) after 48 h (Figure 3C).

Plasmids containing the E1A promoter and E1A coding sequence were engineered to generate an E1A-luciferase fusion transcription cassette. This vector was then further modified to contain four binding sites to mir-122 to allow *in vivo* imaging of E1A expression (Figure 1). Following hydrodynamic delivery of equimolar amounts of both vectors, expression from the plasmid producing the E1A-luciferase fusion protein (with no microRNA sites) was much lower than from the equivalent pCMV vector, probably reflecting relatively weak activity of the E1A promoter in murine cells, however the inclusion of four microRNA sites within this plasmid again mediated a significant decrease in expression (86-fold after 8 h, *P* = 0.01, Figure 3B). It was noticeable that luciferase expression from the fusion protein decreased more rapidly with time than from the pCMV-driven vectors, perhaps reflecting the ability of E1A to negatively regulate its own promoter (Figure 3C).

### Mir-122 binding sites do not affect adenovirus activity in mir-122–negative cells *in vitro* and *in vivo*

**Figure 4:**
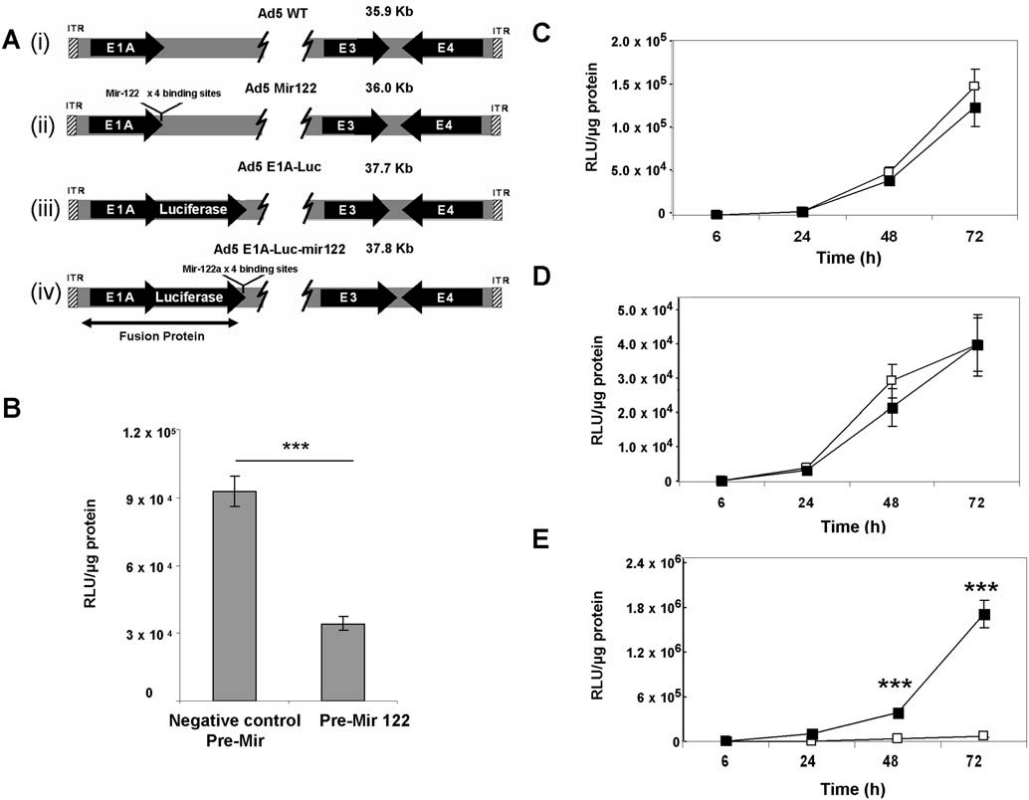
Regulation of E1A expression. (A) Structures of viruses engineered and used in this study. (i) Ad5-WT (ii) Four tandem repeats of binding sites for mir-122 were inserted into the 3′ UTR of E1A. (iii) Luciferase coding sequence was inserted into Ad5 to generate a fusion with the E1A coding sequence. (iv) Four tandem repeats of binding sites for mir-122 were inserted into 3′ UTR of luciferase in the virus shown in iii. (B) A549 cells were seeded at 5×10^4^ cells per well and transfected with pre-mir122 (Ambion) or pre-mir negative control (Ambion). Immediately following transfection Ad-E1A-Luc-mir122 was added at 10 vp/cell in 450 µl DMEM media (10% FCS). 18 h later, 30 pmol/well of pre-cursor mir122 and negative control precursor microRNAs were added to each well in addition to the 500 µl described above. Luciferase readings were performed at 24 h (p = <0.0005). (C–E) Time course of luciferase expression of Ad5-E1A-Luc (solid squares) and Ad5-E1A-Luc-mir (open squares) in mir-122-negative OVCAR3 (C) and A549 (D) cells, and in mir-122-positive Huh7 cells (E) in vitro. (*** P<0.0005).

Adenoviruses containing E1A-luciferase fusion constructs on a background of wild-type Ad5 (Figure 4(iii) and 4(iv)) were used to infect mir-122-negative A549 and OVCAR-3 cell lines *in vitro*. Luciferase activity from both Ad-E1A-Luc (containing no mir-122 binding sites) and Ad-E1A-Luc-mir122 (containing 4 mir122 binding sites) increased slowly between 8 and 24 h and then showed a more rapid rise that was sustained up to at least 72 h (Figure 4B and 4C). This profile of luminescence may reflect initial transcription from the input viral genomes that increases rapidly following viral genomic replication. The microRNA insertion into the 3′ UTR did not affect the profile of luciferase expression in these cells, suggesting the modification did not influence the stability of mRNA encoding the E1A-luciferase fusion protein, nor did it inhibit virus replication in these mir-122-negative cells.

To ascertain whether the microRNA insertion would also be inactive in mir-122- negative cells *in vivo*, viruses (1×10^10^ v.p.) were injected subcutaneously in Balb/C mice (n = 3) and animals were imaged after 24 h. Results demonstrated no significant difference between the expression from the two viruses (data not shown) suggesting no effects of the microRNA at the subcutaneous site.

### MicroRNA binding sites decrease activity of Ad-E1A-luc-mir122 virus in mir122–positive cells *in vitro* and *in vivo*

Adenoviruses encoding the E1A-luciferase protein with and without four microRNA binding sites were used to infect a monolayer of the mir122 positive cell line Huh7. E1A-luciferase expression was monitored by luminometry from 6 h to 72 h post-infection (Figure 4E). Luciferase expression from the Ad5-E1A-Luc showed a small but significant rise between 0 and 24 h (reaching 1.1×10^5^ RLU/µg protein) and then increased rapidly, rising to 1.7×10^6^ RLU/µg protein by 72 h. This suggests E1A transcription and replication proceeded similarly to the situation in A549 and OVCAR cell lines. In contrast, Ad5-E1A-Luc-mir122 virus showed significantly less luciferase expression at all time points, reaching only 6.3×10^4^ RLU/µg after 72 h (*P* = 0.0001 for both 48 and 72 h). The differential in luciferase expression between the viruses with and without microRNA binding sites increased over time, suggesting decreased genome replication of Ad5-E1A-Luc-mir122 compared to Ad5-E1A-Luc. In order to confirm that this differential in luciferase expression was due to mir-122 knockdown of E1A, a precursor RNA mimic of mir-122 (Ambion) was introduced into A549 cells to simulate hepatocyte expression. Ad-E1A-Luc-mir122 and either the mir122 pre-cursor, or negative control pre-mir (Ambion) were added to cells and luciferase readings performed after 24 h. Results showed that the introduction of the pre-mir122 reduced luciferase, and therefore E1A, expression from 9.2×10^4^ RLU (negative control pre-mir) to 3.4×10^3^ RLU (*P* = 0.0001, Figure 4B).

**Figure 5:**
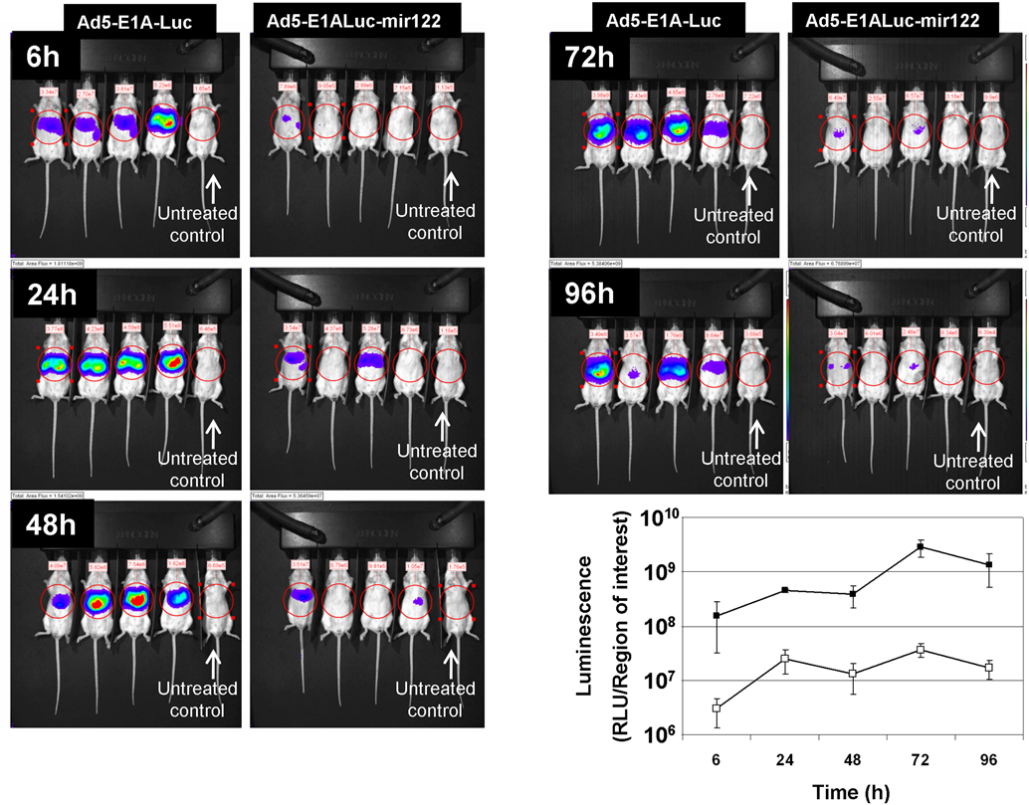
Luciferase transgene expression following intravenous administration of reporter viruses to mice *in vivo*. Groups of four mice were administered intravenously 5×10^10^ virus particles of Ad5-E1A-Luc (left hand group of each pair of images) or Ad5-E1A-Luc-mir122 (right hand group of each pair) and luminescence was quantified using an Ivis100 imaging system after 6 h–96 h. The mouse on the right of all images is an untreated control, mock injected with luciferin for background levels. Images within pairs can be directly compared, although the scaling is different between time points (see scale bars for details). The graph summarises the expression profile as a function of time.

To assess the in vivo activity of these viruses and to observe the effects of time on E1A expression over 96 h, 5×10^10^ vp of Ad5-E1A-Luc and Ad5-E1A-Luc-mir122 were injected intravenously into Balb/C mice. Animals were imaged at 6, 24, 48, 72 & 96 h (Figure 5). After 6 h, Ad-E1A-Luc showed a luminescence signal of 1.6×10^8^ RLU whilst Ad-E1A-Luc-mir122 showed only 3.0×10^6^, a differential of 52-fold. Interestingly, the signal from the Ad5-E1A-Luc treated mice increased by 2.5×10^9^ RLU between 48 and 72 h (Figure 5, time course) possibly reflecting a wave of virus replication. At the same time the microRNA regulated virus showed only a relatively small increase (a rise of 3.4×10^7^ RLU). After 96 h the differential expression between the viruses with and without microRNA sites had reached 80 fold.

### MicroRNA regulation of wild-type Ad5 (Ad5WT) reduces genomic replication and hepatic toxicity

**Figure 6:**
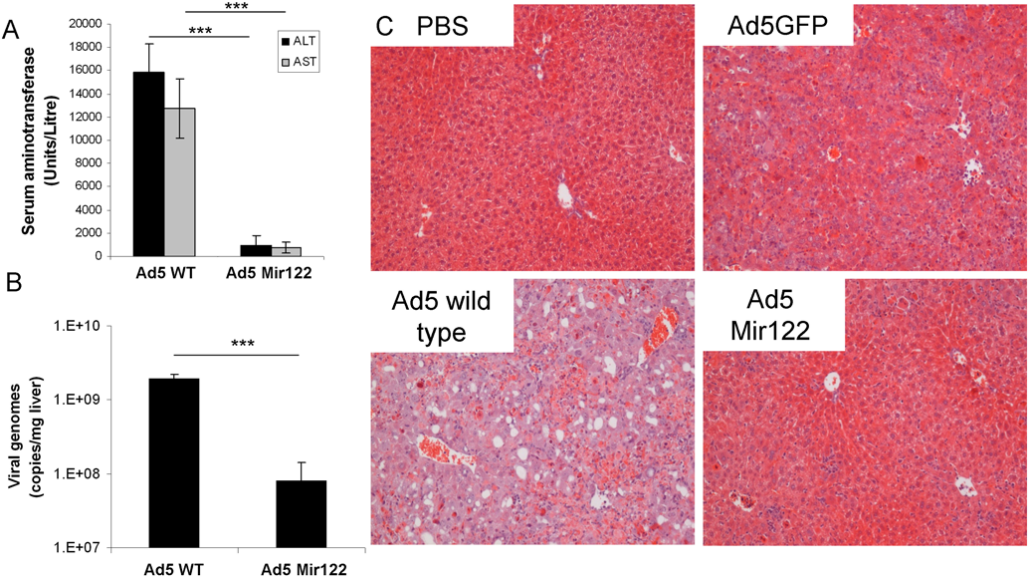
Assessment of hepatotoxicity of wild-type Ad5 modified with microRNA binding sites. (A) Measurement of serum ALT (black bars) and AST (grey bars) 72 h following intravenous administration of 5×10^10^ viral particles of wild-type Ad5 and Ad5-mir122. Analysis was performed as described in the Methods section. (B) Adenovirus genomes in murine liver were measured by real time PCR 72 h following intravenous administration of 5×10^10^ viral particles of wild-type Ad5 WT and Ad5-mir122, as described in the Methods section. (C) Assessment of liver histology. The left liver lobe from each mouse was immersed in 10% buffered formalin overnight at room temperature, embedded in wax and sectioned using a vibratome. Sections were stained with haematoxylin and eosin and analysed by light microscopy at ×40 magnification. Mice were treated with PBS, non-replicating E1, E3-deleted Ad5 expressing GFP (Ad5-GFP), wild-type Ad5, or wild-type Ad5 modified to contain 4 mir-122 binding sites, as indicated. (*** P<0.0005).

In order to assess the effects on hepatic toxicity of including mir-122 binding sites within wild-type adenovirus, 5×10^10^ v.p of Ad5WT and Ad5-mir122 were injected intravenously to Balb/C mice. One mouse in the study which received Ad5WT became hunched and immobile, and was sacrificed after 60 h with visible hepatic pathology. Remaining mice were exsanguinated under anaesthesia 72 h post-injection and blood was allowed to clot. Serum from both groups was tested for Alanine Aminotransferase (ALT) levels and Aspartate Aminotransferase (AST) to assess hepatic damage. Mice administered wild-type Ad5 showed significantly increased ALT levels (90 times higher than control mice treated with PBS, *P* = 0.0001; Figure 6A) suggesting substantial liver damage had occurred. Mice administered Ad5-mir122 showed approximately 15-fold less serum ALT (5 times normal) demonstrating that less liver toxicity had occurred with this virus. AST readings demonstrated similar results with a 17 fold decrease in AST in serum from mice administered Ad5-mir122 compared to serum from mice receiving Ad5WT (*P* = 0.0002, Figure 6A). To evaluate viral replication and tissue damage, livers were divided for histological analysis and QPCR. Livers taken from mice administered wild-type Ad5 showed an average of 2×10^9^ genomes/mg liver (wet weight; Figure 6B). In the total liver this represents approximately 60-fold more genome copies compared to the total amount of virus originally injected, suggesting significant genome replication. In contrast, livers from mice administered Ad5-mir122 showed only 8×10^7^ virus genomes/mg liver, representing less than a doubling compared to the input dose (*P* = 0.0001 for Ad5-mir122 compared to Ad5WT). These data confirm that the microRNA suppression of E1A is capable of significantly reducing replication of the virus genome in murine liver *in vivo*.

Histological analysis showed a dramatic difference between animals administered wild-type Ad5 and those administered Ad5-mir122. Wild-type Ad5 induced vacuolation, haemorrhaging and abnormal nuclear morphology, while livers from mice administered Ad5-mir122 showed very little pathology, with some mice showing no aberrant morphology in any liver section (Figure 6C). Histological images of liver from a mouse administered 5×10^10^ vp of a non-replicating adenoviral vector are presented for comparison, showing similar or slightly greater liver pathology than was induced by Ad5-mir122.

The maximum tolerated dose of Ad5WT given i.v. is reported as about 1×10^9^ PFU [16], and this was confirmed in studies using nude mice bearing HepG2 human hepatocellular carcinoma xenografts (data not shown). Animals were found to tolerate higher levels of Ad-mir122 (6×10^10^ v.p., 9×10^9^ PFU) with only mild weight loss, although when this dose of Ad-mir122 was administered on two consecutive days, all mice were showing signs of virus-related toxicity by day 4 following the first injection. These mice were put down and the livers demonstrated macroscopic signs of viral liver damage. It therefore appears that, in tumour bearing animals, the maximum tolerated dose of Ad-Mir122 lies between 6×10^10^ and 1.2e10^11^v.p/mouse (9×10^9^–1.8×10^10^ pfu).

## Discussion

Molecular engineering of replicating viruses to avoid pathology whilst maintaining potency in therapeutic sites would provide an important new platform for design of viral vaccines and oncolytic treatments. In this study we explored the possibility of achieving this using a DNA virus, wild-type Ad5, engineered to avoid its major toxicity in murine liver by including four binding sites for hepatocyte-specific mir-122 within the 3′UTR of E1A.

To measure E1A expression non-invasively we introduced a luciferase coding region 3′ to the E1A coding region of wild-type virus in order to produce a contiguous E1A-luciferase expression cassette, where E1A splicing would produce a series of E1A-luciferase fusion proteins. This novel virus (including a modified version containing 4 mir-122 binding sites in the E1A 3′ UTR) produced strong luciferase activity *in vitro* and *in vivo* that reported E1A protein levels clearly, enabling non-invasive real-time assessment of protein translation including the effects of virus genome replication. Measuring E1A protein in this way is a more reliable indicator of microRNA activity than measuring E1A mRNA, since microRNA regulation is known to affect protein translation via multiple pathways. However, given that our microRNA target sites are precisely complementary to mir-122 it is likely that argonaut 2 -mediated RNA cleavage is responsible for the majority of the knockdown observed. While the presence of the luciferase sequence slightly decreased the rate of cell killing *in vitro*, compared to the corresponding virus without luciferase, a complete cytopathic effect was still achieved in permissive cells after one extra day. This suggests that the fusion proteins retain all essential E1A functions. This is perhaps unsurprising given that E1A protein has been shown to still operate despite significant deletions and insertions, lacking both enzyme activity and significant secondary structure [17].

Wild-type Ad5 is normally capable of an abortive genome replication cycle in murine liver *in vivo*, where it mediates considerable and sometimes lethal hepatotoxicity [16],[18]. It was unclear whether microRNA regulation could successfully control Ad5, since the DNA genome is not a direct target for microRNA recognition and it is known that even small amounts of E1A translation can lead to genomic replication, which will then provide a template for more transcription providing a greater challenge for microRNA control. Nevertheless, although E1A production in mir-122-positive Huh7 cells *in vitro* was decreased only about 95% following introduction of 4 mir-122-binding into the E1A-luciferase reporter virus, *in vivo* luciferase imaging suggested a greater suppression of E1A expression by mir-122, showing a 50-fold differential after 6 h that rose to 80-fold after 96 h. This may reflect a higher expression of mir-122 in murine hepatocytes *in vivo* than in human Huh7 cells. To complement the E1A reporter luciferase data, hepatic replication and toxicity was also assessed using wild-type Ad5 and compared with a ‘wild-type’ modified to contain four mir-122 sites (Ad-mir122). After 72 h the serum ALT was decreased 15-fold for the microRNA-containing version compared to wild-type, hepatic morphology showed far less evidence of toxicity (most sections appearing normal) and the number of viral genomes found in liver was decreased by a factor of 25. These findings are consistent with those using the E1A-luciferase reporter viruses, and suggest that inclusion of the mir-122-binding sites had a dramatic effect on hepatic activity and toxicity of the virus. It is worth noting that in this study the viruses were applied at dose *in vivo* (5×10^10^ vp/mouse), well above the lethal dose for wild-type Ad5 [16], hence this regulatory strategy appears capable of controlling the activity of significant quantities of virus. Also of note is the ability of mir-122 in mouse liver to tightly regulate the very high levels of E1A-luciferase fusion protein achieved following hydrodynamic plasmid delivery (Figure 3), some 10-fold higher than those shown by the viruses. This suggests that the doses of virus used in this study do not even come close to exceeding the regulatory capacity of mir-122. When even higher virus doses were applied, the maximum tolerated dose of Ad5-mir122 was estimated at between 6×10^10^ and 1.2e10^11^v.p/mouse (9×10^9^–1.8×10^10^ pfu), and such doses presumably allow the virus to break through regulation by hepatic mir-122. Nevertheless these doses are high, affording a range of doses where the virus may be applied therapeutically.

A similar approach, using three microRNA binding sites, has recently been evaluated for regulating activity of CR2-deleted adenovirus in vitro [19]. However, the authors concluded that further attenuation was required in order to prevent CPE in Huh7 cells. The superior performance of the virus reported here may reflect the presence of four microRNA binding sites (rather than three) in the 3′ UTR although it is also possible that Huh7 cells have insufficient mir-122 to achieve the level of virus control seen in primary hepatocytes.

MicroRNA-based virus regulation strategies should find a variety of applications in biotechnology. Their small size (an individual site is typically 22 bp) allows insertion of multiple binding sites, recognising diverse microRNAs, without compromising virus packaging efficiencies. In addition the small insertion size and typical proximity to essential virus genes and regulatory regions (e.g the E1A poly A signal) decreases the likelihood of propagating deletions. Hence a range of stable and versatile agents may be produced using this approach.

Tissue-selective abrogation of virus replication to prevent unwanted pathology should find important applications in cancer virotherapy and also in the production of a new generation of attenuated vaccines for viral diseases. For example, introduction of binding sites for mir122 into the Hepatitis A, B or Hepatitis E genome should prevent replication in hepatocytes and abrogate the main viral toxicity, whilst maintaining infection and possible replication at other cellular sites. Such an approach could yield an important new range of therapeutic vaccines, with applications across the broad sphere of viral diseases.

## Materials and Methods

### Ethics statement

All animal experimentation was performed in accordance with the terms of UK Home Office guidelines and the UKCCCR Guidelines for the Welfare of Animals in Experimental Neoplasia.

### Engineering of microRNA–regulated luciferase reporter plasmids

Luciferase reporter plasmids sensitive to mir-122 were prepared by introducing concatamers of binding sites for mir-122 (4 or 8 sense or 4 antisense binding sites) into the 3′UTR of the luciferase transcription cassette. A CMV-driven luciferase-expressing plasmid vector pCIKLux (a kind gift from Dr Deborah Gill) was cleaved with NotI, oligonucleotides were annealed at 95°C, cooled and ligated into dephosphorylated vector. This produced vectors pCMV-Luc-mir (shown in Figure 1), pCMV-Luc-mir122X8 and pCMV-Luc-mir122anti, together with the control (hereafter referred to as pCMV-Luc) which contained no mir-122 binding sites.

The coding region for the C terminal half of E1A was PCR amplified using Accuprime PFX (Invitrogen) and primers (forward ATT ATA AGA TCT GGA TAG CTG TGA CTC CGG TCC TTC, reverse TAT TCC ATG GAT GGC CTG GGG CGT TTAC) using a plasmid containing wild-type Ad5 as template. These primers introduced unique BglII and Nco1 restriction sites to the 5′ and 3′ termini respectively. The purified PCR product was cleaved with BglII and Nco1 and cloned into pCMV-Luc and pCMV-Luc-mir described above, using the same enzymes, producing a fusion between the C terminal half of E1A and luciferase, including zero or four microRNA sites in the 3′ UTR. These products were subcloned using PshA1 and Hpa1 into a plasmid pAd5-Kpn1 (produced by restriction of wild-type Ad5, see below) to produce plasmids (pE1A-Luc and pE1ALuc-mir122) in which E1A was C-terminally fused to the luciferase coding sequence. The overall scheme of plasmid cloning is shown in Figure 1.

### Cloning of microRNA–regulated wild-type Ad5

Wild-type Ad5 plasmid containing kanamycin resistance (a kind gift from Dr Reuben Hernandez) was cleaved with BstZ17I and recircularised by blunt ended ligation. This vector (Ad5-BstZ17I) was then further cleaved and re-ligated using Kpn1 to increase the number of unique restriction sites available for further cloning. This vector is referred to as Ad5-Kpn1. The 4 microRNA binding sites for mir122 were PCR amplified from pCMV-Luc-mir (described above) to introduce Dra1 sites to each end. The purified PCR product was cleaved with Dra1 and blunt end ligated into Ad5-Kpn1 which was cleaved with Hpa1 in the E1A 3′ UTR. Insertion of microRNA binding sites downstream of E1A was confirmed by DNA sequencing. Ad5-Kpn1-mir122 was reconstituted to Ad5-BstZ17I using the Kpn1 gel-extracted fragment from Ad5-BstZ17I. To generate full size adenovirus genome Ad5-BstZ17I-mir122 was cleaved with BstZ17I, dephosphorylated and subject to homologous recombination with full size wild-type Ad5 ampicillin resistant vector (a kind gift from Dr Peter Searle) and selected on kanamycin. Insertion of microRNA binding sites was confirmed by sequence analysis. Restriction digestion of the resulting vector confirmed full size adenovirus had been recovered.

### Cloning of microRNA–regulated luciferase reporter based on wild-type Ad5

pE1A-Luc-mir and pE1A-Luc (which are modified forms of Ad5-Kpn1 described above) were reconstituted to Ad5-BstZ17I using the Kpn1 gel-extracted fragments from Ad5-BstZ17I. To generate full size adenovirus genome Ad5-BstZ17I-E1ALuc-mir122 and Ad5-BstZ17I-E1ALuc were cleaved with BstZ17I, dephosphorylated and subject to homologous recombination with full size wild-type Ad5 vector (a kind gift from Dr Peter Searle) and selected on kanamycin. Insertion of microRNA binding sites and luciferase was confirmed by sequence analysis. Restriction digests of the resulting vectors confirmed full size adenoviruses had been recovered. Genomic structures and sizes of the viruses are shown in Figure 4A.

### Adenovirus preparations

All adenoviruses were grown in A549 cells, purified by double banding in CsCl gradients with benzonase treatment after the first banding. Viral particle (vp) number was determined by measuring DNA content using a modified version of the PicoGreen assay (Invitrogen, Paisley, UK) [20]. TCID_50_ calculated with the KÄRBER statistical method [21] was used to estimate the adenovirus titer (TCID_50_ units/ml) and corrected to determine plaque forming units/ml (pfu/ml). Adenovirus preparations characteristics are as follows: Ad5 wild-type: 1.13×10^12^ vp/ml, 1.98×10^11^ pfu/ml and particle∶infectivity (P∶I) ratio of 5.6; Ad5-mir122: 1.29×10^12^ vp/ml, 2.01×10^11^ pfu/ml and particle∶infectivity (P∶I) ratio of 6.4. All virus preparations were screened for endotoxin and verified negative prior to use.

### Maintenance of cell lines

Human hepatocellular carcinoma HUH7 cells, A549 lung carcinoma cells, OVCAR3 ovarian cancer cells and HEK293 human embryonic kidney cells were obtained from the European Collection of Cell Cultures (Porton Down, UK), and maintained in DMEM with 10% foetal bovine serum (FBS) (PAA Laboratories, Yeovil, UK) including penicillin (25 U/ml) and streptomycin (10 mg/ml).

### Luciferase expression assays *in vitro*

Cells were seeded in triplicate in 12 well plates. After 24 h plasmid DNA (0.5 µg) was added to 50 µl of HBS buffer and mixed with 2.5 µl DOTAP reagent (Roche) also in 50 µl sterile HBS. The complex was incubated at room temperature for 30 min. 100 µl of transfection mixture was added to each well and incubated at 37°C for 4 h. Cells were washed with PBS and incubated with DMEM containing 2% FBS. 24 h following transfection media were removed and 150 µl reporter cell lysis buffer (Promega) was added to the cells. Cells were then frozen at −80°C for >1 h before thawing. Luciferin (25 µl) (Promega, Southampton, UK) was added to 25 µl aliquots of cell lysate and relative luminescence was measured by luminometry (Lumat LB 9507, Berthold Technologies, Redbourn, UK).

### Pre-mir 122 transfection

A549 cells were seeded at 5×10^4^ cells per well and incubated overnight. Pre-mir122 (Ambion) and pre-mir negative control (Ambion) were re-suspended to 50 µM and then further diluted 10 fold. 3 µl per well of this dilution of each pre-mir was added to 22 µl Opti-MEM medium (Invitrogen). 2 µl per well of NeoFx transfection reagent (Ambion) was added to 23 µl Opti-MEM solution. Pre-mir/Opti-MEM was mixed with the NeoFx/Optimem and allowed to complex for 10 minutes. A549 cells were washed with PBS and the transfection mixture added to cells at a total volume of 50 µl. Total amount of pre-mir is 15 pmol/well. Immediately following transfection Ad-E1A-Luc-mir122 was added at 10 vp/cell in 450 µl DMEM media (10% FCS). 18 h later, 30 pmol/well of pre-cursor mir122 and negative control pre-cursor microRNAs were added to each well in addition to the 500 µl described above. Luciferase readings were performed at 24 h.

### Real time (quantitative) PCR (Q–PCR) for Ad5

The Q-PCR methodology for measurement of adenoviral particles has been previously described [22]. Viral DNA from infected cell or tissue samples were extracted using a mammalian genomic DNA miniprep kit (Sigma). Reactions were performed using Applied Biosystems master mix following the manufacturer's protocol. The cycles were as follows: 94°C 10 min; 40 times (94°C 30 s, 60°C 1 min). Primers sequences for targeting Ad5 fiber are: FW- TGG CTG TTA AAG GCA GTT TGG (Ad5 32350–32370 nt) and RV- GCA CTC CAT TTT CGT CAA ATC TT (Ad5 32433–32411 nt) and the TaqMan probe- TCC AAT ATC TGG AAC AGT TCA AAG TGC TCA TCT (Ad5 32372–32404 nt), dual labeled at the 5′ end with 6-carboxyfluorescein and the 3′ end with 6-carboxytetramethylrhodamine. The results were analyzed with the Sequence Detection System software (Applied Biosystems). Standard curves for tissues and cells were prepared by spiking samples of cell lysate or tissue homogenate with serial dilutions of known concentrations of virus particles and then extracting and analysing each sample separately by Q-PCR as described above.

### Measurement of Serum Alanine Aminotransferase (ALT) and Aspartate Aminotransferase (AST)

Blood was taken from mice by cardiac puncture and allowed to clot (15 min, room temperature) and spun at 1200 g for 10 min. Serum was isolated and immediately frozen at −20°C). Samples of thawed serum (5 µl) were added to ALT reagent (995 µl, Microgenics) or AST reagent (995 µl, Microgenics) in a 1 ml quartz cuvette, incubated at 37°C and the change in absorbance (340 nm) per minute was monitored. Units of ALT and AST activity were calculated according to the manufacturer's instructions.

### Assessment of hepatic expression of plasmids in mice

Plasmids were administered by hydrodynamic injection (0.8 pmole/mouse, using a 10% body volume of PBS administered over 5–10 s with a 27 gauge needle) into the tail veins of Balb/c mice. Non-invasive measurement of luminescence was performed after 8, 24 and 48 h using an IVIS 100 system (Xenogen, MA) under isofluorane anaesthetic. Luciferin was administered by intraperitoneal injection (15.8 mg/ml in PBS, 100 µl/mouse) 4 min prior to imaging. Flux levels were analyzed with Living Image Software (Xenogen, MA).

### Evaluation of the activity of adenoviruses containing microRNA binding sites *in vivo*

Clodronate was a gift of Roche Diagnostics GmbH, Mannheim, Germany. It was encapsulated in liposomes as described previously [23]. Viruses were administered intravenously (unless otherwise indicated) and all animals were pretreated with bisphosphonate liposomes (100 µl/mouse, obtained from Dr Nico van Rouijen) 24 h before. For imaging expression of E1A encoded within replication-competent Ad5, E1A-luciferase reporter viruses with and without 4 binding sites for mir122 (Ad5-E1A-luc and Ad5-E1A-luc-mir122) were injected intravenously to Balb/c mice (5×10^10^ v.p./mouse). Animals were imaged after 6, 24, 48, 72 and 96 h as described above.

To study the ability of mir-122-binding sites included within wild-type A5 to decrease hepatic replication of virus genomes and tissue damage, 5×10^10^ v.p./mouse of Ad5WT and Ad5-mir122 were injected i.v. Animals were monitored twice daily and sacrificed after 72 h for measurement of genome replication (by QPCR) and assessment of pathology (by histological analysis).

### Histology

The left liver lobe from each mouse was immersed in 10% buffered formalin overnight at room temperature, embedded in wax and sectioned using a vibratome. Sections were stained with haematoxylin and eosin and analysed by light microscopy at ×40 magnification.

### Statistical analysis

*In vitro* data are expressed as the mean of 3 replicates ±standard deviation unless otherwise stated. In vivo data are expressed as the mean of four replicates ±standard deviation, except using the plasmid pCMV-Luc-Mir for which n = 3. Significance was evaluated using t-test and denoted on the graphs as * P<0.05, ** P<0.005, *** P<0.0005.
